# Aging and Western Diet Synergistically Impair Hepatic Thyroid Hormone Signaling to Promote Metabolic Dysfunction‐Associated Steatotic Liver Disease (MASLD) in Mice

**DOI:** 10.1111/acel.70600

**Published:** 2026-06-23

**Authors:** Xinru Zhang, Madhulika Tripathi, Chun Ting Goh, Chee Seng Chan, Anita Boelen, Paul M. Yen, Brijesh Kumar Singh, Eveline Bruinstroop

**Affiliations:** ^1^ Endocrine Laboratory, Department of Laboratory Medicine, Amsterdam Gastroenterology, Amsterdam UMC University of Amsterdam Amsterdam the Netherlands; ^2^ Research Institute Amsterdam Gastroenterology Endocrinology & Metabolism (AGEM), Amsterdam UMC Amsterdam the Netherlands; ^3^ Laboratory of Hormonal Regulation, Cardiovascular and Metabolic Disorders Program Duke–NUS Medical School Singapore Singapore; ^4^ National Heart Centre Singapore Singapore Singapore; ^5^ Department of Endocrinology and Metabolism Amsterdam UMC, University of Amsterdam Amsterdam the Netherlands

**Keywords:** aging, deiodinase, hepatocyte senescence, MASLD, resmetirom, thyroid hormone, Western diet

## Abstract

Metabolic dysfunction‐associated steatotic liver disease (MASLD) is primarily driven by a Western‐style diet and exacerbated with aging, yet underlying mechanisms remain unclear. Given the essential role of thyroid hormone (TH) in MASLD progression, we hypothesized that impaired intrahepatic TH action during aging promotes MASLD progression and severity of MASH with fibrosis. We evaluated hepatic TH metabolism in young (18–24 weeks) and old (108–120 weeks) C57BL/6J mice fed either a normal chow diet (NCD) or a Western diet with fructose (WDF) for 8 weeks. Liver histology, metabolic parameters, inflammatory and fibrotic markers, intrahepatic thyroxine (T4) and triiodothyronine (T3) concentrations, and activities of deiodinase enzymes (Dio1 and Dio3) were measured. Additionally, an in vitro hepatocyte senescence model using AML12 cells was employed to assess age‐related alterations in deiodinase expression and the therapeutic efficacy of resmetirom (an FDA‐approved thyromimetic). Aging and WDF synergistically exacerbated hepatic inflammation and fibrosis, accompanied by significant reductions in intrahepatic T4 and T3. Aging markedly decreased Dio1 activity, which converts T4 to active T3, whereas WDF partially restored Dio1 in old mice. Conversely, Dio3 activity, responsible for TH inactivation, increased with age but exhibited age‐dependent differential responses to WDF, findings mirrored in senescent hepatocytes. Notably, resmetirom significantly reduced senescence markers, inhibited senescence‐associated secretory phenotype (SASP) genes, inflammasome activation, endoplasmic reticulum (ER) stress, and activated autophagy. Collectively, our findings demonstrate that aging and stress by a Western‐style diet synergistically impair hepatic TH signaling, accelerating MASLD progression. Furthermore, resmetirom improved hepatic senescence, highlighting its potential therapeutic repurposing for aging‐associated hepatic pathologies, including MASLD.

## Graphical abstract

Aging and Western diet synergistically impair hepatic thyroid hormone signaling through decreased Dio1 and increased Dio3 activity, depleting intrahepatic T4 and T3 in senescent hepatocytes. This thyroid hormone deficit drives inflammation, fibrosis, and accelerated MASLD progression. Resmetirom, a TRβ agonist, reverses cellular senescence markers, reduces senescence‐associated secretory phenotype (SASP) expression, and restores autophagy, identifying thyroid hormone signaling as a promising therapeutic target for age‐associated metabolic liver disease.

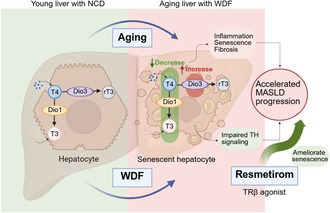

Metabolic dysfunction‐associated steatotic liver disease (MASLD) is the hepatic manifestation of obesity and diabetes, which now occurs in 38% of the adult population and projected to be about 55% by the year 2040 (Younossi et al. 2025). Its prevalence increases among the aging population and represents a significant public health challenge (Estes et al. 2018). The median age of MASLD population was 50 years in 2015 and is projected to reach 55 by 2030 (He et al. 2024). Thus, as the population ages, MASLD‐related liver disease incidence and mortality is rising. Aging‐associated factors, such as chronic inflammation and cellular senescence, may exacerbate MASLD progression, although mechanisms linking aging and liver dysfunction remain poorly understood (Du et al. 2025). Thyroid hormone (TH) critically regulates hepatic lipid metabolism, and even mild hypothyroidism increases MASLD risk (Ratziu et al. 2025). TH actions are mediated primarily through binding of the bioactive triiodothyronine (T3) to the nuclear thyroid hormone receptors (TRα and TRβ), with TRβ particularly essential in hepatic metabolism (Ratziu et al. 2025). It has been shown that hepatic TRβ activity is a critical determinant of MASLD progression in patients (Kendall et al. 2023), and selective TRβ analogues (e.g., resmetirom) have therapeutic benefit by enhancing hepatic fatty acid utilization and lipid clearance (Harrison et al. 2019, 2024).

Hepatic TH availability is regulated by Type 1 deiodinase (Dio1), which converts thyroxine (T4, the prohormone) to T3 and clears reverse T3 (rT3, the inactive metabolite). Type 3 deiodinase (Dio3) inactivates T3 and T4 (Bruinstroop et al. 2023; Sinha et al. 2025). We previously demonstrated that hepatic Dio1 activity increases during early metabolic dysfunction‐associated steatohepatitis (MASH) (Bruinstroop et al. 2021), potentially serving a protective role through enhanced T3‐mediated lipid metabolism, and declines during MASH progression (Bruinstroop et al. 2018). However, Dio1 activity declines with aging, reducing hepatic T3 production and elevating serum rT3 in older individuals (van den Beld et al. 2005). This age‐related decline in hepatic TH activation may exacerbate MASLD severity. Notably, a Western diet with fructose (WDF) independently reduces hepatic TH concentrations in young mice (Bruinstroop et al. 2021). We therefore hypothesized that aging exacerbates diet‐induced hepatic TH dysregulation, which potentially increases MASLD susceptibility in older individuals. To this end, we first evaluated hepatic effects of aging and diet by comparing young (18–24 weeks) and old (108–120 weeks) C57BL/6J mice fed either a normal chow diet (NCD) or a Western diet with fructose (WDF) for 8 weeks (Figure 1A and Figure S1). An overview of the results of the 2‐way ANOVA (Age, Diet, Interaction) is provided in Table S1. WDF induced significant hepatic steatosis in both age groups, demonstrated by elevated lipid accumulation histologically and liver triglyceride (TG) content (Figure 1B,C). Mild increases with no significant changes in steatosis (Liver TG) occur between young WDF and old WDF mice. Consistent with age‐related chronic low‐grade inflammation, old mice on NCD exhibited significantly higher baseline expression of some inflammatory and fibrotic genes (*Il1b* and *Acta2*), further exacerbated by WDF with increased expression of all inflammatory and fibrosis genes (Young WDF vs. Old WDF) (Figure 1D,E). Hepatic hydroxyproline (HPA, a collagen derivative) was significantly elevated in old WDF mice compared to young WDF (Figure 1F). We performed histological assessment and indeed confirmed an increased NAS score (NAFLD Activity Score) and increased perivenular and pericellular fibrosis in Old‐WDF liver (Figure S2 and Table S2). Additionally, hepatic senescence markers increased significantly with age (Young NCD vs. Old NCD and Young WDF vs. Old WDF) with no additional effect of WDF (Figure 1G). This shows that old mice have an increased inflammatory and fibrotic phenotype compared to young WDF mice. We next evaluated whether these changes are associated with changes in intrahepatic thyroid hormone metabolism.

**FIGURE 1:**
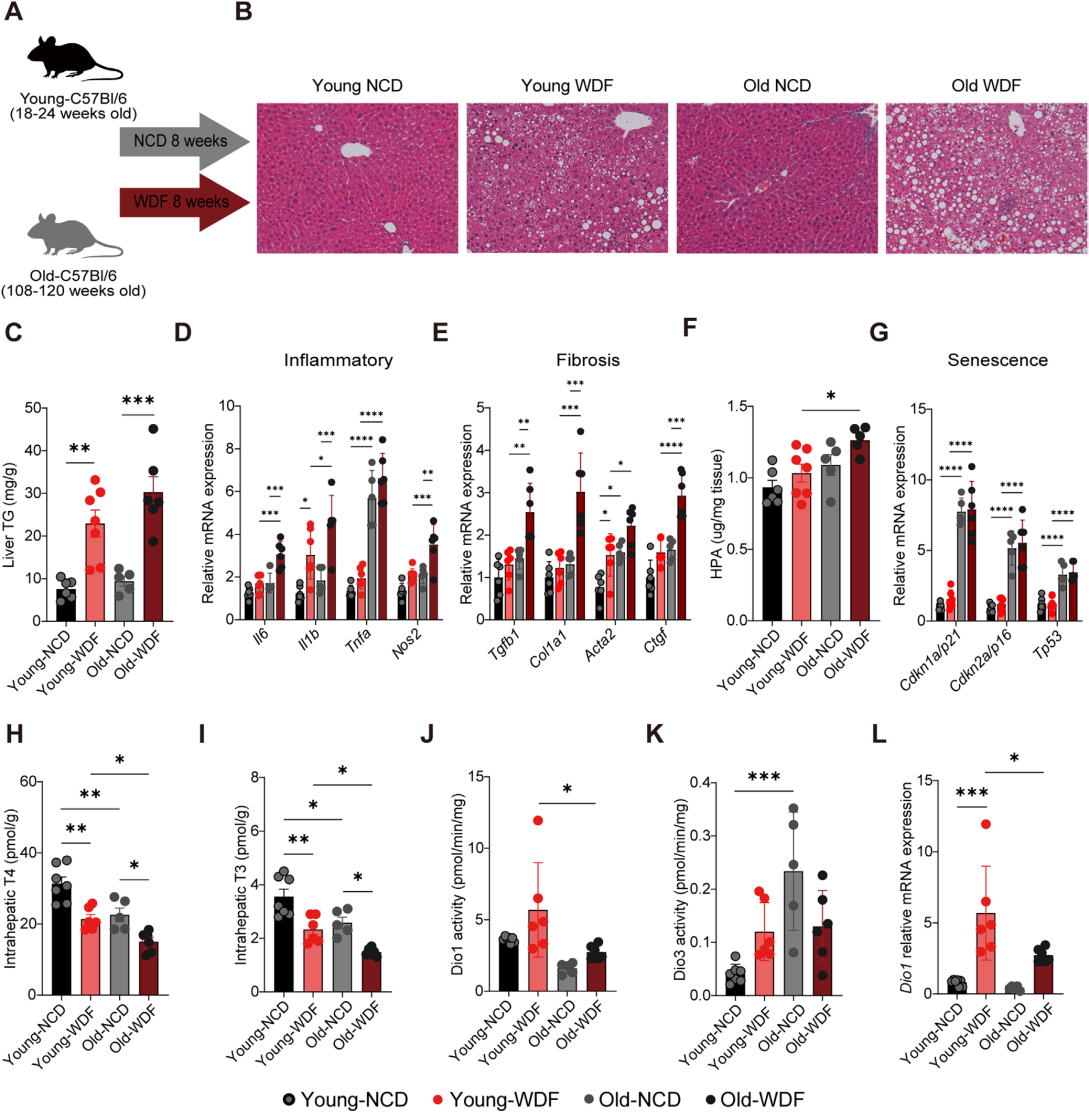
Intrahepatic thyroid hormone (TH) concentrations declined with aging and MASLD progression, with reduced Dio1 and increased Dio3 activities. (A) Schematic showing administration protocol for Western Diet with 15% fructose in the drinking water (WDF) for 8 weeks compared to Normal Chow Diet (NCD) in the young and old mice (*n* = 7–8 per group). (B) Representative liver histological images (10 × scale bar = 100 μm). (C) Liver triglyceride (TG) content. (D, E) Hepatic mRNA expression of inflammatory and fibrotic markers. (F) Hepatic hydroxyproline (HPA) content. (G) Senescence‐related mRNA expression. (H, I) Intrahepatic T4 and T3 concentrations. (J, K) Dio1 and Dio3 activity. (L) *Dio1* relative mRNA expression. Statistical analysis was performed by two‐way ANOVA with subsequent Tukey's multiple comparison. Data were presented as mean ± SD. *p < 0.05, **p < 0.01, ***p < 0.001, ****p < 0.0001.

Intrahepatic T4 was significantly reduced by both aging (*p* < 0.001) and WDF (*p* < 0.0001) with no interaction effect (Figure 1H). Similarly, intrahepatic bioactive T3 also markedly declined with age (*p* < 0.01) with further reduction by WDF (*p* < 0.001) (Figure 1I). Mechanistically, age‐related TH disruption correlated with altered deiodinase expression. Dio1 activity (*p* < 0.01) and expression (*p* < 0.05) significantly decreased with age. As shown previously (Bruinstroop et al. 2021), WDF increases Dio1 mRNA expression (*p* < 0.0001) and activity (*p* < 0.05), albeit there was only a modest compensatory increase in old mice (Figure 1J,L). Conversely, Dio3 activity increased with age (*p* < 0.05); however, there is a significant interaction effect where there is a trend toward increase in young mice and decrease in old mice (Age × Diet *p* < 0.05) (Figure 1K). No changes were observed in thyroid hormone transporters *MCT8* and *OATP1c1* mRNA (Figure S3). The increase in Dio3 in MASLD has previously been shown to occur after liver injury and MASLD by activation of a repair‐related sonic hedgehog signaling pathway, and aging has shown to dampen this pathway (Bohinc et al. 2014; Maeso‐Díaz et al. 2022). We indeed showed that in young mice hedgehog pathways genes were upregulated in young mice after WDF, which did not occur in older mice, which may explain the differential effect on Dio3 and increased fibrosis (Figure S4 and Table S3). Collectively, these results demonstrate that aging and diet synergistically compromise hepatic TH homeostasis. This could potentially exacerbate MASLD severity during aging, as it was previously shown that liver‐specific knockdown of Dio1 by siRNA injection led to increased severity of MASLD (Bruinstroop et al. 2021). Furthermore, reversing intrahepatic hypothyroidism by treatment with levothyroxine or a TRβ agonist has been shown to be beneficial (Bruinstroop et al. 2018; Harrison et al. 2024).

Hepatocytes are the main cell type within the liver. Hepatocyte senescence has been shown to be related to MASLD stage, diabetes mellitus and liver‐related outcomes (Aravinthan et al. 2013; Du et al. 2025). We therefore next investigated whether hepatocyte senescence is associated with disrupted hepatic TH metabolism using an in vitro hepatocyte senescence model (AML12 cells) as described previously (Tripathi et al. 2020). Although this model represents an accelerated stimulus compared to the chronic, low‐grade oxidative stress that characterizes in vivo hepatic aging, we previously compared senescent AML12 cells to livers from 108‐ to 128‐week‐old mice directly and demonstrated that this model recapitulates the molecular and metabolic signatures of aged liver, including fuel switching toward glycolysis, impaired mitochondrial oxidative phosphorylation, decreased autophagy, and increased mTOR signaling (Singh et al. 2020). Senescent AML12 cells expressed classical cell‐cycle arrest markers (*p21*, *p16*, and *p53*) and a pronounced senescence‐associated secretory phenotype (SASP), including elevated cytokines *IL‐1β* and *IL‐6* (Figure 2A). Importantly, senescent hepatocytes mirrored aged liver phenotypes, showing reduced *Dio1* and increased *Dio3* expression relative to controls (Figure 2B). Gene expression of *p21* and *Dio1* were significantly negatively correlated whereas *p21* and *Dio3* were positively correlated (Figure 2C and Figure S5), supporting a direct association between cellular senescence and impaired TH metabolism.

**FIGURE 2:**
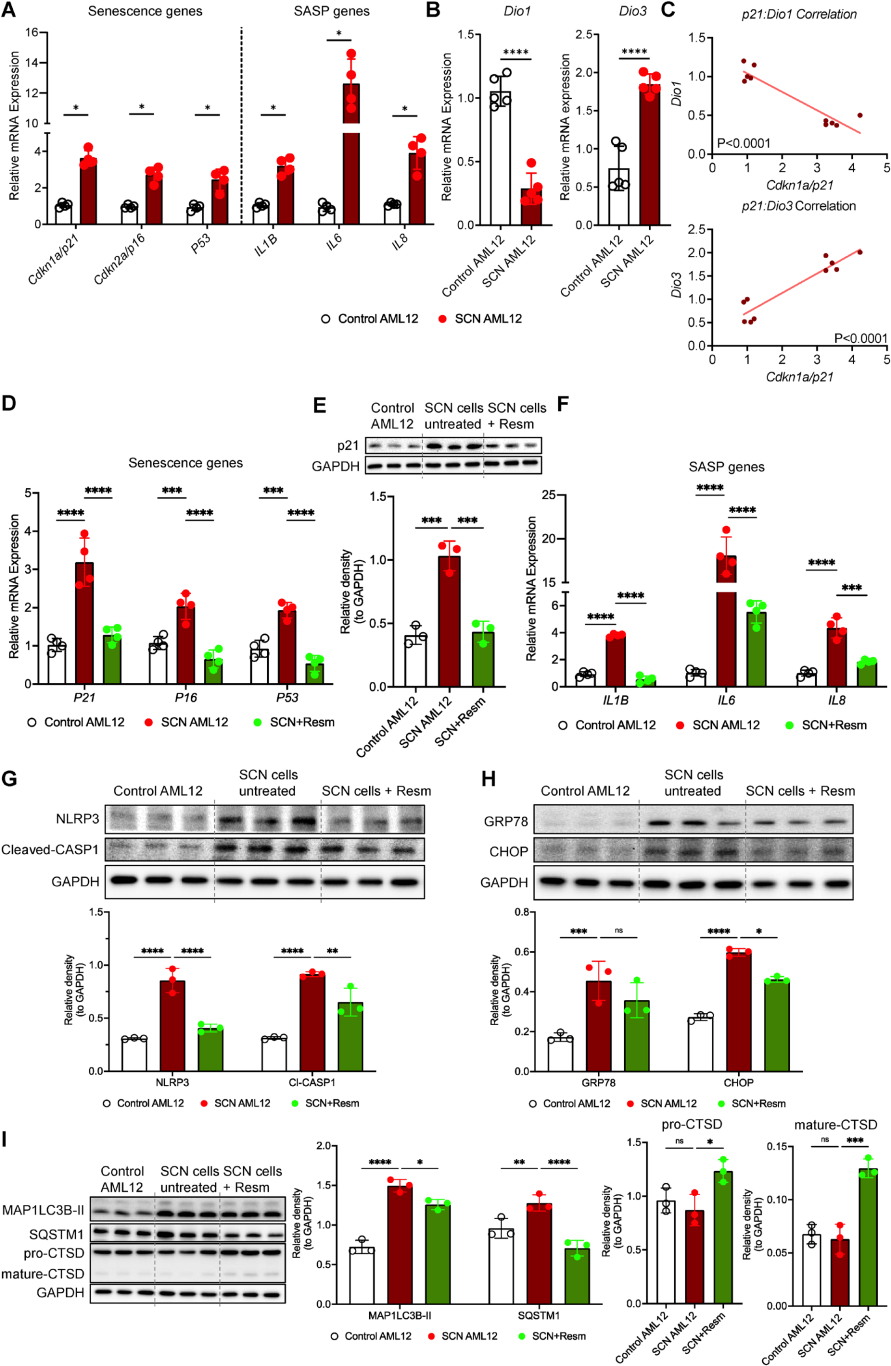
Dio1 expression was reduced in hepatocellular senescence and resmetirom improved senescence‐associated stress and inflammatory phenotype. (A) Hepatic mRNA expression of senescence (SCN) and senescence‐associated secretory phenotype (SASP) genes. (B) *Dio1* and *Dio3* mRNA expression. (C) Linear regression and correlation analysis for SCN gene *p21* (SCN marker) with *Dio1* and *Dio3* mRNA expression. (D) *p21*, *p16*, and *p53* mRNA expression, and (E) p21 protein levels (Western blot, with quantification). (F) Resmetirom effects on *IL‐1β*, *IL‐6*, and *IL‐8* mRNA expression. (G) NLRP3 and cleaved caspase‐1 (CASP1) protein levels (Western blot, with quantification). (H) BiP/GRP78 and CHOP protein levels (Western blot, with quantification). (I) Autophagy markers: MAP1LC3B‐II, p62/SQSTM1, pro‐CTSD, and mature CTSD protein levels (Western blot, with quantification). All data are representative of ≥ 3 independent experiments. Statistical testing was performed using unpaired Student's *t*‐test or one‐way ANOVA with subsequent Tukey's multiple comparison. Data were presented as mean ± SD. ns = no significance, *p < 0.05, **p < 0.01, ***p < 0.001, ****p < 0.0001.

Finally, we tested whether enhancing hepatic TH signaling could ameliorate senescence‐induced dysfunction in our in vitro model. Senescent AML12 cells were treated with resmetirom, a selective TRβ agonist clinically registered for MASH treatment (Harrison et al. 2024). Resmetirom acts as a ligand for the hepatic TR which, similar to T3, leads to increased expression of Dio1 (Hönes et al. 2022). Strikingly, resmetirom treatment markedly reduced senescence markers, including *p21*, *p16*, and *p53* mRNA (Figure 2D) and p21 protein levels (Figure 2E). Additionally, SASP cytokines *IL‐1β*, *IL‐6*, and *IL‐8* were significantly diminished following resmetirom treatment (Figure 2F). Key inflammasome proteins NLRP3 and cleaved caspase‐1, those play key roles in the release of inflammatory factors that contribute to the SASP in senescent cells (Wiggins and Clarke 2019), also significantly declined (Figure 2G). Moreover, resmetirom effectively attenuated endoplasmic reticulum stress, indicated by reduced CHOP expression (Figure 2H). We also performed analysis of Pcna, a marker for the loss of proliferative capacity occurring during senescence, and found no changes after resmetirom treatment (Figure S6). The data suggest that resmetirom may not affect cell proliferation, whereas it may prevent senescence induction and associated ER stress. Resmetirom was also found to improve autophagy flux, characterized by decreased levels of MAP1LC3B‐II and reduced accumulation of autophagy substrate p62/SQSTM1, accompanied by increased levels of pro‐CTSD and mature CTSD (Figure 2I). Autophagy impairment leads to enhanced SASP expression (Rastaldo et al. 2020). Moreover, defective autophagy during aging contributes to increased ER stress and inflammation, as functional autophagy is crucial for maintaining protein homeostasis and reducing ER‐stress‐driven senescence (Ghosh et al. 2016; Ma et al. 2024). Together, these findings show that treatment with a thyromimetic attenuates cellular senescence markers and SASP expression but also mitigates inflammasome activation, endoplasmic reticulum stress, and restores impaired autophagy flux. This should be further tested in animal or human models that also recapitulate all cell types within the liver and further investigate whether thyromimetics could also prevent senescence and improve other markers of senescence (Pcna). To our knowledge, this is the first study directly linking hepatocyte senescence to altered intrahepatic TH homeostasis. This association could be caused by cytokines such as *IL‐6* and *IL‐1β*, known SASP components, which have been shown to reduce Dio1 activity and induce Dio3 expression (Bruinstroop et al. 2023; Wajner et al. 2011). Alternatively, age‐related changes in TH metabolism (decreased Dio1, lower T3) may aggravate senescence. This possibility is supported by the in vitro data showing that enhancing TH signaling via resmetirom can significantly reverse multiple senescence‐related pathways, supporting its potential therapeutic utility against age‐related hepatic disorders, such as MASLD.

## Author Contributions

X.Z. and M.T. analyzed data and wrote the manuscript, E.B. and B.K.S. designed, performed experiments and collected data, C.T.G. and C.C.S. assisted with experiments, E.B, A.B., P.M.Y., and B.K.S. supervised the project. All authors read and approved the final manuscript.

## Funding

This work is supported by Ministry of Health (MOH), National Medical Research Council (NMRC), Singapore, Grant Number: MOH‐000319 (MOH‐OFIRG19may‐0002) (B.K.S.); Duke/Duke‐NUS Research Collaboration Pilot Project Award, Grant Number: Duke/Duke‐NUS/RECA(Pilot)/2022/0060 (B.K.S.); KBrFA, Grant Number: Duke‐NUS‐KBrFA/2023/0075 (to B.K.S.). X.Z. was funded by the Chinese Scholarship Council (CSC) [NO. 202307720074].

## Conflicts of Interest

E.B. received speaker fees from Madrigal and consultancy fees from Aligos.

## Supporting information


**Appendix S1:** Aging cell author checklist.

**Figure S1:** Young (18–24 weeks) and old (108–120 weeks) male mice liver metabolic parameters. (A) Body weight, (B) Fat mass, (C) liver index (liver weight to body weight ratio; LW/BW) and (D) fasting glucose. Statistical analysis was performed by two‐way ANOVA with subsequent Tukey's multiple comparison. Data were presented as mean ± SD. *p < 0.05, **p < 0.01, ***p < 0.001, ****p < 0.0001.
**Figure S2:** Histological assessment of MASLD severity in young and aged mice on NCD or WDF. (A) NAFLD Activity Score (NAS) components and total score in Young‐NCD, Young‐WDF, Old‐NCD, and Old‐WDF mice (*n* = 5 per group). (B) Representative liver Picrosirius Red staining images showing collagen deposition (10× magnification, scale bar = 100 μm). Data in (A) are presented as mean ± SD with individual data points. Statistical analysis by Kruskal–Wallis test was performed followed by Dunn's multiple comparison test. *p < 0.05, **p < 0.01.
**Figure S3:** Thyroid hormone transporters *Mct8* and *Oatp1c1* expressions did not change in aging and diet. (A) *Dio3* mRNA expressions in young and old mice fed WDF or NCD model. (B, C) *Mct8* and *Oatp1c1* mRNA expressions in young and old mice fed WDF or NCD model. Thyroid hormone transporters monocarboxylate transporter 8: *Mct8*, organic anion‐transporting polypeptide 1c1: *Oatp1c1*. Statistical testing was performed using a two‐way ANOVA with subsequent Tukey's multiple comparison. Data were presented as means ± SD.
**Figure S4:** Young (18–24 weeks) and old (108–120 weeks) male mice liver hedgehog pathway genes. *Ihh*, *Gli3*, *Angpt1*, *Sox9*, *Pcna* mRNA expressions in young and old mice fed WDF or NCD model. Statistical analysis was performed by two‐way ANOVA with subsequent Tukey's multiple comparison. Data were presented as mean ± SD. *p < 0.05, **p < 0.01, ***p < 0.001, ****p < 0.0001, ns = no significance.
**Figure S5:** Hepatic Dio1 activity negatively correlated with p21 expression in vivo. Linear regression and correlation analysis between hepatic Dio1 enzyme activity (pmol/min/mg protein) and *Cdkn1a (p21)* mRNA expression in liver samples from young and aged mice fed NCD or WDF.
**Figure S6:** Resmetirom did not rescue the senescence‐induced reduction of Pcna in AML12 cells. (A) Relative Pcna mRNA expression in control AML12 cells, senescent AML12 cells (SCN), and senescent AML12 cells treated with resmetirom (SCN + Resm) (*n* = 3 per group). (B) Representative Western blot of PCNA protein with GAPDH as loading control (upper panel), and quantification of PCNA protein normalized to GAPDH (lower panel) (*n* = 3 per group). Data are presented as mean ± SD with individual data points. Statistical analysis was performed by one‐way ANOVA followed by Tukey's multiple comparison test. **p < 0.01; ns = not significant.

**Table S1:** Two‐way ANOVA results for animal model parameters.

**Table S2:** Kruskal–Wallis test followed by Dunn's multiple comparison test results for NAS.

**Table S3:** Two‐way ANOVA with subsequent Tukey's multiple comparison results for hedgehog pathway genes.

## Data Availability

The data that support the findings of this study are available on request from the corresponding author. The data are not publicly available due to privacy or ethical restrictions. The completed Aging Cell author checklist is provided in Appendix S1.
