# Supplementary material for: Aging and Western Diet Synergistically Impair Hepatic Thyroid Hormone Signaling to Promote Metabolic Dysfunction‐Associated Steatotic Liver Disease (MASLD) in Mice
Source: Aging Cell. 2026 Jun 23;25(7):e70600. doi: 10.1111/acel.70600 (PMC13288151; doi:10.1111/acel.70600)
Supplement: Supplementary file 1 — Appendix S1: Aging cell author checklist. [file ACEL-25-e70600-s004.docx]

**AGING CELL AUTHOR CHECKLIST**. ***Authors should submit this checklist together with their manuscript. Please ensure that you have read the Author Guidelines in detail before submission.***

| **Title** | **Aging and Western diet synergistically impair hepatic thyroid hormone signaling to promote metabolic dysfunction-associated steatotic liver disease (MASLD) in mice** | | | | | | |
| --- | --- | --- | --- | --- | --- | --- | --- |
| **Authors** | **Xinru Zhang^1,2,#^, Madhulika Tripathi^3#^, Chun Ting Goh^3^, Chan Chee Seng^3^, Anita Boelen^1,2^, Paul M. Yen^3^,** **Brijesh Kumar Singh^3*^, Eveline Bruinstroop^2,4,*^** | | | | | | |
| **Manuscript Type** | **Short Communication** | | | | | | |
| **Total Character Count (including spaces)^1^** | **9,078** | | | | | | |
| **Word count of Summary^2^** | **247** | | | | | | |
| **Number of papers cited in the References^3^** | **20** | | | | | | |
| **Listing of all Tables (Table1, Table 2 etc)^4^** | **-** | | | | | | |
|  |  | | | | | | |
|  |  | | | | | | |
| **Figure specifications (please complete one row per figure)^5^**  ***Figure no.*** | Colour  ***(yes/no)*** | Greyscale  ***(yes/no)*** | Black and white  ***(yes/no)*** | Single column (80mm)  ***(yes/no)*** | Double column (180mm)  ***(yes/no)*** | Size of figure at full scale  (mm x mm)  ***(insert details)*** | Smallest font size used in the figure at full scale (minimum 6pt)  ***(insert***  ***details)*** |
| **1** | **yes** | **no** | **no** | **no** | **yes** | **180mm x 184.91mm** | **7pt** |
| **2** | **yes** | **no** | **no** | **no** | **yes** | **177.64mm x 275mm** | **7pt** |
|  |  |  |  |  |  |  |  |
|  |  |  |  |  |  |  |  |
|  |  |  |  |  |  |  |  |
|  |  |  |  |  |  |  |  |
|  |  |  |  |  |  |  |  |

**^1^** The maximum character count allowed is 50,000 (incl. spaces) for Primary Research Papers and Reviews, 10,000 for Short Takes.

**^2^** Summary should not exceed 250 words.

**^3^** Primary Research Papers can contain a maximum of two tables. If more are needed they should replace some of the Figures or can be placed in the Supporting Information.

**^4^** A maximum of 45 references is allowed for Primary Research Papers and 20 references for Short Takes.

**^5^** A Primary Research Paper may contain up to 6 figures and a Short Take up to 2 figures. Authors are encouraged to provide figures in the size they are to appear in the journal and at the specifications given.
