# Supplementary material for: Aging and Western Diet Synergistically Impair Hepatic Thyroid Hormone Signaling to Promote Metabolic Dysfunction‐Associated Steatotic Liver Disease (MASLD) in Mice
Source: Aging Cell. 2026 Jun 23;25(7):e70600. doi: 10.1111/acel.70600 (PMC13288151; doi:10.1111/acel.70600)
Supplement: Supplementary file 2 — Figure S1: Young (18–24 weeks) and old (108–120 weeks) male mice liver metabolic parameters. (A) Body weight, (B) Fat mass, (C) liver index (liver weight to body weight ratio; LW/BW) and (D) fasting glucose. Statistical analysis was performed by two‐way ANOVA with subsequent Tukey's multiple comparison. Data were presented as mean ± SD. *p < 0.05, **p < 0.01, ***p < 0.001, ****p < 0.0001. Figure S2: Histological assessment of MASLD severity in young and aged mice on NCD or WDF. (A) NAFLD Activity Score (NAS) components and total score in Young‐NCD, Young‐WDF, Old‐NCD, and Old‐WDF mice (n = 5 per group). (B) Representative liver Picrosirius Red staining images showing collagen deposition (10× magnification, scale bar = 100 μm). Data in (A) are presented as mean ± SD with individual data points. Statistical analysis by Kruskal–Wallis test was performed followed by Dunn's multiple comparison test. *p < 0.05, **p < 0.01. Figure S3: Thyroid hormone transporters Mct8 and Oatp1c1 expressions did not change in aging and diet. (A) Dio3 mRNA expressions in young and old mice fed WDF or NCD model. (B, C) Mct8 and Oatp1c1 mRNA expressions in young and old mice fed WDF or NCD model. Thyroid hormone transporters monocarboxylate transporter 8: Mct8, organic anion‐transporting polypeptide 1c1: Oatp1c1. Statistical testing was performed using a two‐way ANOVA with subsequent Tukey's multiple comparison. Data were presented as means ± SD. Figure S4: Young (18–24 weeks) and old (108–120 weeks) male mice liver hedgehog pathway genes. Ihh, Gli3, Angpt1, Sox9, Pcna mRNA expressions in young and old mice fed WDF or NCD model. Statistical analysis was performed by two‐way ANOVA with subsequent Tukey's multiple comparison. Data were presented as mean ± SD. *p < 0.05, **p < 0.01, ***p < 0.001, ****p < 0.0001, ns = no significance. Figure S5: Hepatic Dio1 activity negatively correlated with p21 expression in vivo. Linear regression and correlation analysis between hepatic Dio1 enzyme activity [file ACEL-25-e70600-s005.zip › acel70600-sup-0003-FigureS1-S6@Supporting information_clean vision.docx]

**Aging and Western diet synergistically impair hepatic thyroid hormone signaling to promote metabolic dysfunction-associated steatotic liver disease (MASLD) in mice**

Xinru Zhang^1,2,#^, Madhulika Tripathi^3#^, Chun Ting Goh^3^, Chan Chee Seng^3^, Anita Boelen^1,2^, Paul M. Yen^3^, Brijesh Kumar Singh^3*^, Eveline Bruinstroop^2,4,*^

**Supplementary information**

**Table of contents**

[Supplementary Material and Methods 2](#_Toc230099036)

[*Animals and WDF diet feeding* 2](#_Toc230099037)

[*Cell culture* 2](#_Toc230099038)

[*Deiodinase measurements* 3](#_Toc230099039)

[*Thyroid hormones in tissue* 4](#_Toc230099040)

[*RNA isolation and quantitative real-time PCR* 4](#_Toc230099041)

[*Histological analysis of liver tissue* 5](#_Toc230099042)

[*Statistical analysis* 5](#_Toc230099043)

[Supplementary References 6](#_Toc230099044)

[Supplementary Figure Legend 8](#_Toc230099045)

# **Supplementary Material and Methods**

## *Animals and WDF diet feeding*

All animal procedures were approved by the Singhealth Institutional Animal Care and Use Committee (2020/SHS/1549). Male C57Bl/6J mice, aged 18-24 weeks (Young group) and 108-120 weeks (Old group), were acquired from The Jackson Laboratory (Bar Harbor, Maine, USA). For a duration of 8 weeks, male C57Bl/6J mice in both the Young (n = 7-8 per group) and Old groups (n = 7-8 per group) were housed at a standard temperature of 22 °C and administered a Western diet (D12079B; Research Diets) supplemented with 15% weight/volume fructose (Sigma-Aldrich, 57-48-7) dissolved in their drinking water (WDF) as described earlier (Bruinstroop et al., 2021; Tripathi et al., 2022). Control mice were fed a standard chow diet with normal water (NCD) for the same period (n = 7-8 per group). Body weight and fat mass were measured periodically using EchoMRI, Body Composition Analyzer. Fasting glucose was measured before euthanization after 6 h fasting using hand-held Accu-Chek blood glucose analyzer with strips. Mice were euthanized and tissues were collected. The liver lobe was fixed in 10% buffered formalin for histological analysis or snap-frozen in liquid nitrogen and stored at -80 °C for subsequent analysis.

## *Cell culture*

AML12 cells (ATCC) were passaged alternately in complete growth medium (DMEM: F12 supplemented with 10% FBS, 1×penicillin/streptomycin, and insulin-transferrin-selenium) and basal DMEM medium (with only penicillin/streptomycin) in preparation for H₂O₂ treatment. Upon reaching approximately 50% confluence, the complete growth medium was replaced with prewarmed basal medium containing 1.0 mM H₂O₂ for 1 hour followed by 23 h recovery in complete growth medium. Subsequently, on the second, third, fourth, fifth, and sixth days, the medium was again changed to prewarmed basal medium containing 0.75 mM H₂O₂ for 1 hour each followed by 23 h recovery in complete growth medium, as per the protocol for generating senescent cells from the mouse hepatic cell line AML12 to study hepatic aging (Singh et al., 2020; Tripathi, Yen, & Singh, 2020). Resmetirom (100 µM) or vehicle (DMSO) was added to the cells during 23 h recovery in complete growth medium as shown below.


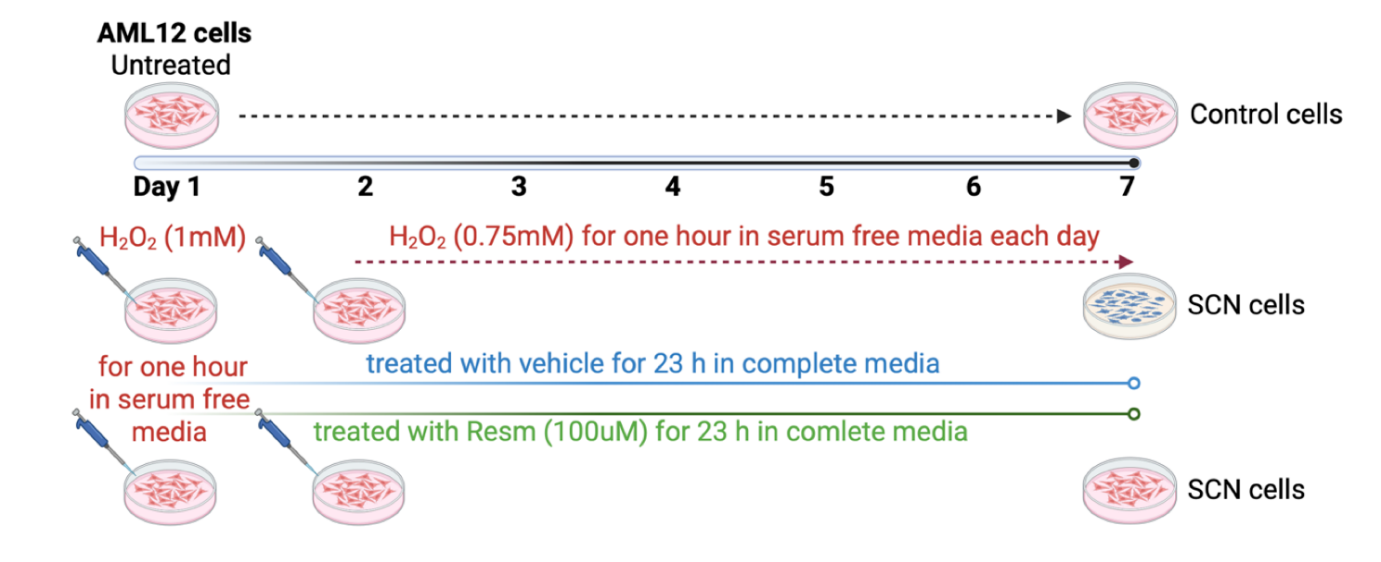


## *Deiodinase measurements*

Liver Dio1 and Dio3 activities were measured as previously described (Boelen et al., 2017; de Vries et al., 2014). Briefly, for deiodinase activity measurements samples were homogenized on ice in phosphate-EDTA (PE) buffer [0.1 M sodium phosphate, 2 mM EDTA (pH 7.2)] and dithiothreitol (DTT). Protein concentration was measured with the Bio-Rad protein assay using bovine serum albumin (BSA) as the standard following the manufacturer’s instructions (Bio-Rad Laboratories, Veenendaal, The Netherlands). Liver Dio1 activity was measured using a [3’5’-^125^I] rT3 tracer. One sample from each group was incubated with 500 μM PTU to inhibit Dio1 activity, serving as a tissue blank. Dio3 activity was measured using a [3’5’-^125^I] T3 tracer. One sample of each group was incubated in the presence of 500 nM unlabelled T3 to inhibit Dio3 activity representing a tissue blank. The radioactivity of the substrate and the product in the eluate was measured online using a Radiometric 150TR flow scintillation analyser (Perkin Elmer, Waltham, MA, USA). Dio1 activity was expressed as pmol generated T2/minute/mg protein. Intra-assay variation is 5.5%. Dio3 activity was expressed as fmol generated T2/minute/mg protein. Intra-assay variation is 5.5%.

## *Thyroid hormones in tissue*

Intrahepatic T3 and T4 concentrations were determined in 25 mg frozen liver tissue with a liquid chromatography mass spectrometry (LC–MS/MS) method as described before (Ackermans, Kettelarij-Haas, Boelen, & Endert, 2012; de Vries et al., 2014).

## *RNA isolation and quantitative real-time PCR*

Total RNA was isolated using the Roche High Pure RNA isolation kit (Roche) according to the manufacturer’s instructions. RNA yield was quantified by the DeNovix nanodrop (DeNovix, Wilmington, USA) and cDNA was synthesized with equal RNA input using the Transcriptor First Strand cDNA synthesis Kit (Roche Molecular Biochemicals, Mannheim, Germany). Several randomly selected samples were also processed without reverse transcriptase during cDNA synthesis to check for genomic DNA contamination. Quantitative PCR was performed using the SensiFAST SYBR No-ROX (Bioline) and the LightCycler480 (Roche Molecular Biochemicals, Mannheim, Germany). Predesigned KiCqStart SYBR Green optimized primers from Sigma-Aldrich (KSPQ12012) were used for RT-qPCR. Quantification was performed using the LC480 conversion (v.2014.1) and LinRegPCR (v.2021.1) software. PCR efficiency of each sample was calculated and samples with a deviation of more than 5% of the mean efficiency value of the assay were excluded. The expression levels of mRNA in the samples were normalized using the geometric mean of reference gene, resulting in relative expression values.

## *Histological analysis of liver tissue*

Liver samples were harvested post-euthanization, fixed in 10% neutral-buffered formalin for 24 hours, and subsequently embedded in paraffin. Serial sections (5 µm thickness) were prepared using a microtome and mounted on glass slides. Sections were deparaffinized, rehydrated through graded alcohols, and stained with hematoxylin and eosin (H&E) for gross histological evaluation. Stained sections were examined under an Olympus light microscope, and representative images were captured at 10 × magnification. Key features associated with MASLD, including hepatic triglyceride (TG), were assessed qualitatively. NAS scoring was performed as described elsewhere (Liang et al., 2014). Picro Sirius Red Stain Kit (Connective Tissue Stain, ab150681, Abcam) was used for Sirius red staining in liver sections as as per the manufacturer’s protocol. Hepatic hydroxyproline (HPA) was measure as described earlier (Widjaja et al., 2019).

## *Statistical analysis*

Analysis was performed on at least three biological replicates, ie. three independent experiments performed on three independent days. All statistical analyses were performed using GraphPad Prism 10.3 (GraphPad Software). Data were presented as mean ± SD. Normal distribution of the data was tested using the Shapiro-Wilk test. In general, for comparing two conditions among groups a two-way ANOVA with subsequent Tukey’s multiple comparison was used to investigate the effect of age, diet and the interaction effect. For the in vitro analysis, an unpaired Student’s t-test was used. All p-values < 0.05 were considered significant.

Supplementary References

Ackermans, M. T., Kettelarij-Haas, Y., Boelen, A., & Endert, E. (2012). Determination of thyroid hormones and their metabolites in tissue using SPE UPLC-tandem MS. *Biomed Chromatogr, 26*(4), 485-490. doi:10.1002/bmc.1691

Boelen, A., van der Spek, A. H., Bloise, F., de Vries, E. M., Surovtseva, O. V., van Beeren, M., . . . Fliers, E. (2017). Tissue thyroid hormone metabolism is differentially regulated during illness in mice. *J Endocrinol, 233*(1), 25-36. doi:10.1530/joe-16-0483

Bruinstroop, E., Zhou, J., Tripathi, M., Yau, W. W., Boelen, A., Singh, B. K., & Yen, P. M. (2021). Early induction of hepatic deiodinase type 1 inhibits hepatosteatosis during NAFLD progression. *Mol Metab, 53*, 101266. doi:10.1016/j.molmet.2021.101266

de Vries, E. M., Eggels, L., van Beeren, H. C., Ackermans, M. T., Kalsbeek, A., Fliers, E., & Boelen, A. (2014). Fasting-induced changes in hepatic thyroid hormone metabolism in male rats are independent of autonomic nervous input to the liver. *Endocrinology, 155*(12), 5033-5041. doi:10.1210/en.2014-1608

Liang, W., Menke, A. L., Driessen, A., Koek, G. H., Lindeman, J. H., Stoop, R., . . . van den Hoek, A. M. (2014). Establishment of a general NAFLD scoring system for rodent models and comparison to human liver pathology. *PLoS One, 9*(12), e115922. doi:10.1371/journal.pone.0115922

Singh, B. K., Tripathi, M., Sandireddy, R., Tikno, K., Zhou, J., & Yen, P. M. (2020). Decreased autophagy and fuel switching occur in a senescent hepatic cell model system. *Aging (Albany NY), 12*(14), 13958-13978. doi:10.18632/aging.103740

Tripathi, M., Singh, B. K., Zhou, J., Tikno, K., Widjaja, A., Sandireddy, R., . . . Yen, P. M. (2022). Vitamin B(12) and folate decrease inflammation and fibrosis in NASH by preventing syntaxin 17 homocysteinylation. *J Hepatol, 77*(5), 1246-1255. doi:10.1016/j.jhep.2022.06.033

Tripathi, M., Yen, P. M., & Singh, B. K. (2020). Protocol to Generate Senescent Cells from the Mouse Hepatic Cell Line AML12 to Study Hepatic Aging. *STAR Protoc, 1*(2), 100064. doi:10.1016/j.xpro.2020.100064

Widjaja, A. A., Singh, B. K., Adami, E., Viswanathan, S., Dong, J., D'Agostino, G. A., . . . Cook, S. A. (2019). Inhibiting Interleukin 11 Signaling Reduces Hepatocyte Death and Liver Fibrosis, Inflammation, and Steatosis in Mouse Models of Nonalcoholic Steatohepatitis. *Gastroenterology, 157*(3), 777-792.e714. doi:10.1053/j.gastro.2019.05.002

Supplementary Figure Legend

**Figure S1. Young (18-24 weeks) and old (108-120 weeks) male mice liver metabolic parameters.** (**A**) Body weight, (**B**) Fat mass, (**C**) liver index (liver weight to body weight ratio; LW/BW) and (**D**) fasting glucose. Statistical analysis was performed by two-way ANOVA with subsequent Tukey’s multiple comparison. Data were presented as mean ± SD. *p < 0.05, **p < 0.01, ***p < 0.001, ****p < 0.0001.

**Figure S2. Histological assessment of MASLD severity in young and aged mice on NCD or WDF.** (**A**) NAFLD Activity Score (NAS) components and total score in Young-NCD, Young-WDF, Old-NCD, and Old-WDF mice (n=5 per group). (**B**) Representative liver Picrosirius Red staining images showing collagen deposition (10× magnification, scale bar = 100 μm). Data in (**A**) are presented as mean ± SD with individual data points. Statistical analysis by Kruskal-Wallis test was performed followed by Dunn's multiple comparison test. *p<0.05, **p<0.01.

**Figure S3. Thyroid hormone transporters *Mct8* and *Oatp1c1* expressions did not change in aging and diet.** (**A**) *Dio3* mRNA expressions in young and old mice fed WDF or NCD model. (**B, C**) *Mct8* and *Oatp1c1* mRNA expressions in young and old mice fed WDF or NCD model. Thyroid hormone transporters monocarboxylate transporter 8: *Mct8*, organic anion-transporting polypeptide 1c1: *Oatp1c1*. Statistical testing was performed using a two-way ANOVA with subsequent Tukey’s multiple comparison. Data were presented as means ± SD.

**Figure S4. Young (18-24 weeks) and old (108-120 weeks) male mice liver hedgehog pathway genes.** *Ihh*, *Gli3*, *Angpt1*, *Sox9*, *Pcna* mRNA expressions in young and old mice fed WDF or NCD model. Statistical analysis was performed by two-way ANOVA with subsequent Tukey’s multiple comparison. Data were presented as mean ± SD. *p < 0.05, **p < 0.01, ***p < 0.001, ****p < 0.0001, ns=no significance.

**Figure S5. Hepatic Dio1 activity negatively correlated with p21 expression in vivo.** Linear regression and correlation analysis between hepatic Dio1 enzyme activity (pmol/min/mg protein) and *Cdkn1a (p21)* mRNA expression in liver samples from young and aged mice fed NCD or WDF.

**Figure S6. Resmetirom did not rescue the senescence-induced reduction of Pcna in AML12 cells.** (**A**) Relative Pcna mRNA expression in control AML12 cells, senescent AML12 cells (SCN), and senescent AML12 cells treated with resmetirom (SCN+Resm) (n=3 per group). (**B**) Representative Western blot of PCNA protein with GAPDH as loading control (upper panel), and quantification of PCNA protein normalized to GAPDH (lower panel) (n=3 per group). Data are presented as mean ± SD with individual data points. Statistical analysis was performed by one-way ANOVA followed by Tukey’s multiple comparison test. **p<0.01; ns=not significant.
