# Supplementary figures and images for: Aging and Western Diet Synergistically Impair Hepatic Thyroid Hormone Signaling to Promote Metabolic Dysfunction‐Associated Steatotic Liver Disease (MASLD) in Mice
Source: Aging Cell. 2026 Jun 23;25(7):e70600. doi: 10.1111/acel.70600 (PMC13288151; doi:10.1111/acel.70600)

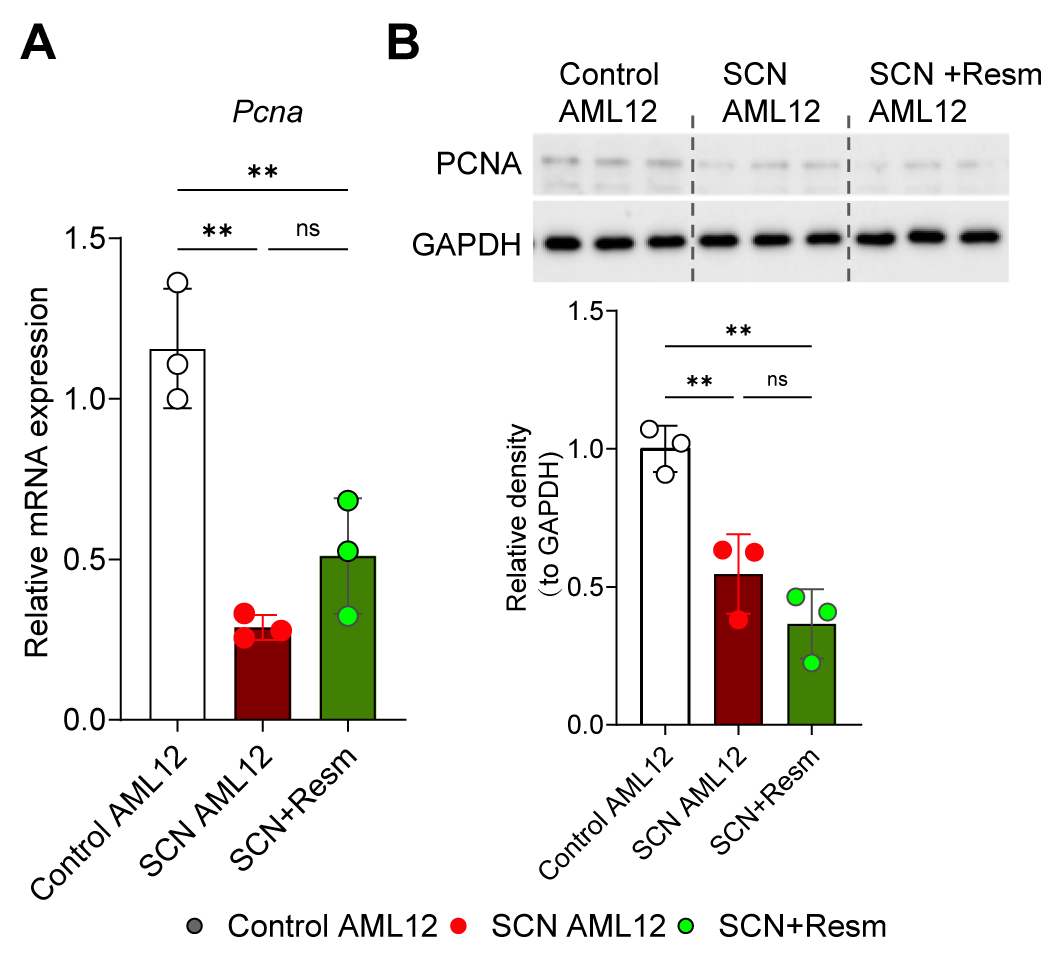

Supplement: Supplementary file 2 — Figure S1: Young (18–24 weeks) and old (108–120 weeks) male mice liver metabolic parameters. (A) Body weight, (B) Fat mass, (C) liver index (liver weight to body weight ratio; LW/BW) and (D) fasting glucose. Statistical analysis was performed by two‐way ANOVA with subsequent Tukey's multiple comparison. Data were presented as mean ± SD. *p < 0.05, **p < 0.01, ***p < 0.001, ****p < 0.0001. Figure S2: Histological assessment of MASLD severity in young and aged mice on NCD or WDF. (A) NAFLD Activity Score (NAS) components and total score in Young‐NCD, Young‐WDF, Old‐NCD, and Old‐WDF mice (n = 5 per group). (B) Representative liver Picrosirius Red staining images showing collagen deposition (10× magnification, scale bar = 100 μm). Data in (A) are presented as mean ± SD with individual data points. Statistical analysis by Kruskal–Wallis test was performed followed by Dunn's multiple comparison test. *p < 0.05, **p < 0.01. Figure S3: Thyroid hormone transporters Mct8 and Oatp1c1 expressions did not change in aging and diet. (A) Dio3 mRNA expressions in young and old mice fed WDF or NCD model. (B, C) Mct8 and Oatp1c1 mRNA expressions in young and old mice fed WDF or NCD model. Thyroid hormone transporters monocarboxylate transporter 8: Mct8, organic anion‐transporting polypeptide 1c1: Oatp1c1. Statistical testing was performed using a two‐way ANOVA with subsequent Tukey's multiple comparison. Data were presented as means ± SD. Figure S4: Young (18–24 weeks) and old (108–120 weeks) male mice liver hedgehog pathway genes. Ihh, Gli3, Angpt1, Sox9, Pcna mRNA expressions in young and old mice fed WDF or NCD model. Statistical analysis was performed by two‐way ANOVA with subsequent Tukey's multiple comparison. Data were presented as mean ± SD. *p < 0.05, **p < 0.01, ***p < 0.001, ****p < 0.0001, ns = no significance. Figure S5: Hepatic Dio1 activity negatively correlated with p21 expression in vivo. Linear regression and correlation analysis between hepatic Dio1 enzyme activity [file ACEL-25-e70600-s005.zip › acel70600-sup-0008-FigureS6-S6@Supplementary_Figure_S6.tif]

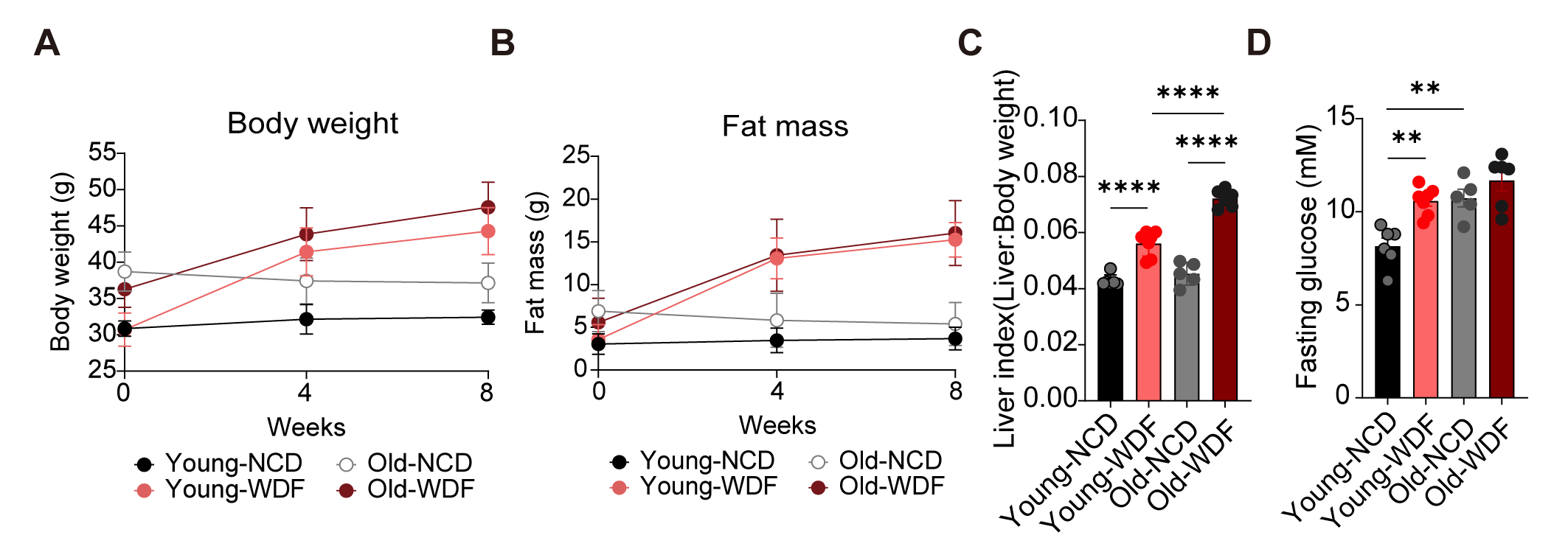

Supplement: Supplementary file 2 — Figure S1: Young (18–24 weeks) and old (108–120 weeks) male mice liver metabolic parameters. (A) Body weight, (B) Fat mass, (C) liver index (liver weight to body weight ratio; LW/BW) and (D) fasting glucose. Statistical analysis was performed by two‐way ANOVA with subsequent Tukey's multiple comparison. Data were presented as mean ± SD. *p < 0.05, **p < 0.01, ***p < 0.001, ****p < 0.0001. Figure S2: Histological assessment of MASLD severity in young and aged mice on NCD or WDF. (A) NAFLD Activity Score (NAS) components and total score in Young‐NCD, Young‐WDF, Old‐NCD, and Old‐WDF mice (n = 5 per group). (B) Representative liver Picrosirius Red staining images showing collagen deposition (10× magnification, scale bar = 100 μm). Data in (A) are presented as mean ± SD with individual data points. Statistical analysis by Kruskal–Wallis test was performed followed by Dunn's multiple comparison test. *p < 0.05, **p < 0.01. Figure S3: Thyroid hormone transporters Mct8 and Oatp1c1 expressions did not change in aging and diet. (A) Dio3 mRNA expressions in young and old mice fed WDF or NCD model. (B, C) Mct8 and Oatp1c1 mRNA expressions in young and old mice fed WDF or NCD model. Thyroid hormone transporters monocarboxylate transporter 8: Mct8, organic anion‐transporting polypeptide 1c1: Oatp1c1. Statistical testing was performed using a two‐way ANOVA with subsequent Tukey's multiple comparison. Data were presented as means ± SD. Figure S4: Young (18–24 weeks) and old (108–120 weeks) male mice liver hedgehog pathway genes. Ihh, Gli3, Angpt1, Sox9, Pcna mRNA expressions in young and old mice fed WDF or NCD model. Statistical analysis was performed by two‐way ANOVA with subsequent Tukey's multiple comparison. Data were presented as mean ± SD. *p < 0.05, **p < 0.01, ***p < 0.001, ****p < 0.0001, ns = no significance. Figure S5: Hepatic Dio1 activity negatively correlated with p21 expression in vivo. Linear regression and correlation analysis between hepatic Dio1 enzyme activity [file ACEL-25-e70600-s005.zip › acel70600-sup-0002-FigureS1-S1@Supplementary_Figure_S1.tif]

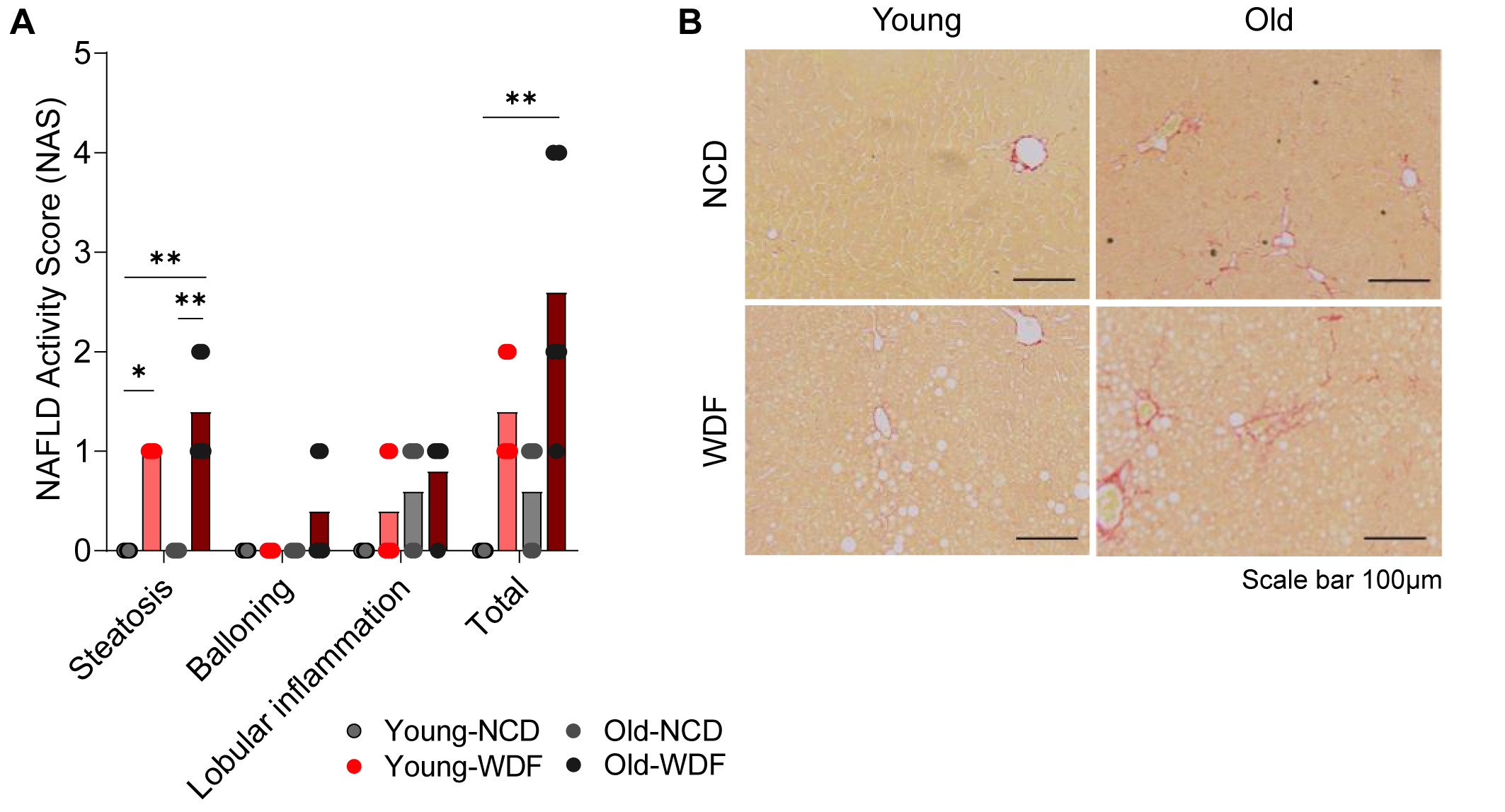

Supplement: Supplementary file 2 — Figure S1: Young (18–24 weeks) and old (108–120 weeks) male mice liver metabolic parameters. (A) Body weight, (B) Fat mass, (C) liver index (liver weight to body weight ratio; LW/BW) and (D) fasting glucose. Statistical analysis was performed by two‐way ANOVA with subsequent Tukey's multiple comparison. Data were presented as mean ± SD. *p < 0.05, **p < 0.01, ***p < 0.001, ****p < 0.0001. Figure S2: Histological assessment of MASLD severity in young and aged mice on NCD or WDF. (A) NAFLD Activity Score (NAS) components and total score in Young‐NCD, Young‐WDF, Old‐NCD, and Old‐WDF mice (n = 5 per group). (B) Representative liver Picrosirius Red staining images showing collagen deposition (10× magnification, scale bar = 100 μm). Data in (A) are presented as mean ± SD with individual data points. Statistical analysis by Kruskal–Wallis test was performed followed by Dunn's multiple comparison test. *p < 0.05, **p < 0.01. Figure S3: Thyroid hormone transporters Mct8 and Oatp1c1 expressions did not change in aging and diet. (A) Dio3 mRNA expressions in young and old mice fed WDF or NCD model. (B, C) Mct8 and Oatp1c1 mRNA expressions in young and old mice fed WDF or NCD model. Thyroid hormone transporters monocarboxylate transporter 8: Mct8, organic anion‐transporting polypeptide 1c1: Oatp1c1. Statistical testing was performed using a two‐way ANOVA with subsequent Tukey's multiple comparison. Data were presented as means ± SD. Figure S4: Young (18–24 weeks) and old (108–120 weeks) male mice liver hedgehog pathway genes. Ihh, Gli3, Angpt1, Sox9, Pcna mRNA expressions in young and old mice fed WDF or NCD model. Statistical analysis was performed by two‐way ANOVA with subsequent Tukey's multiple comparison. Data were presented as mean ± SD. *p < 0.05, **p < 0.01, ***p < 0.001, ****p < 0.0001, ns = no significance. Figure S5: Hepatic Dio1 activity negatively correlated with p21 expression in vivo. Linear regression and correlation analysis between hepatic Dio1 enzyme activity [file ACEL-25-e70600-s005.zip › acel70600-sup-0004-FigureS2-S2@Supplementary_Figure_S2.tif]

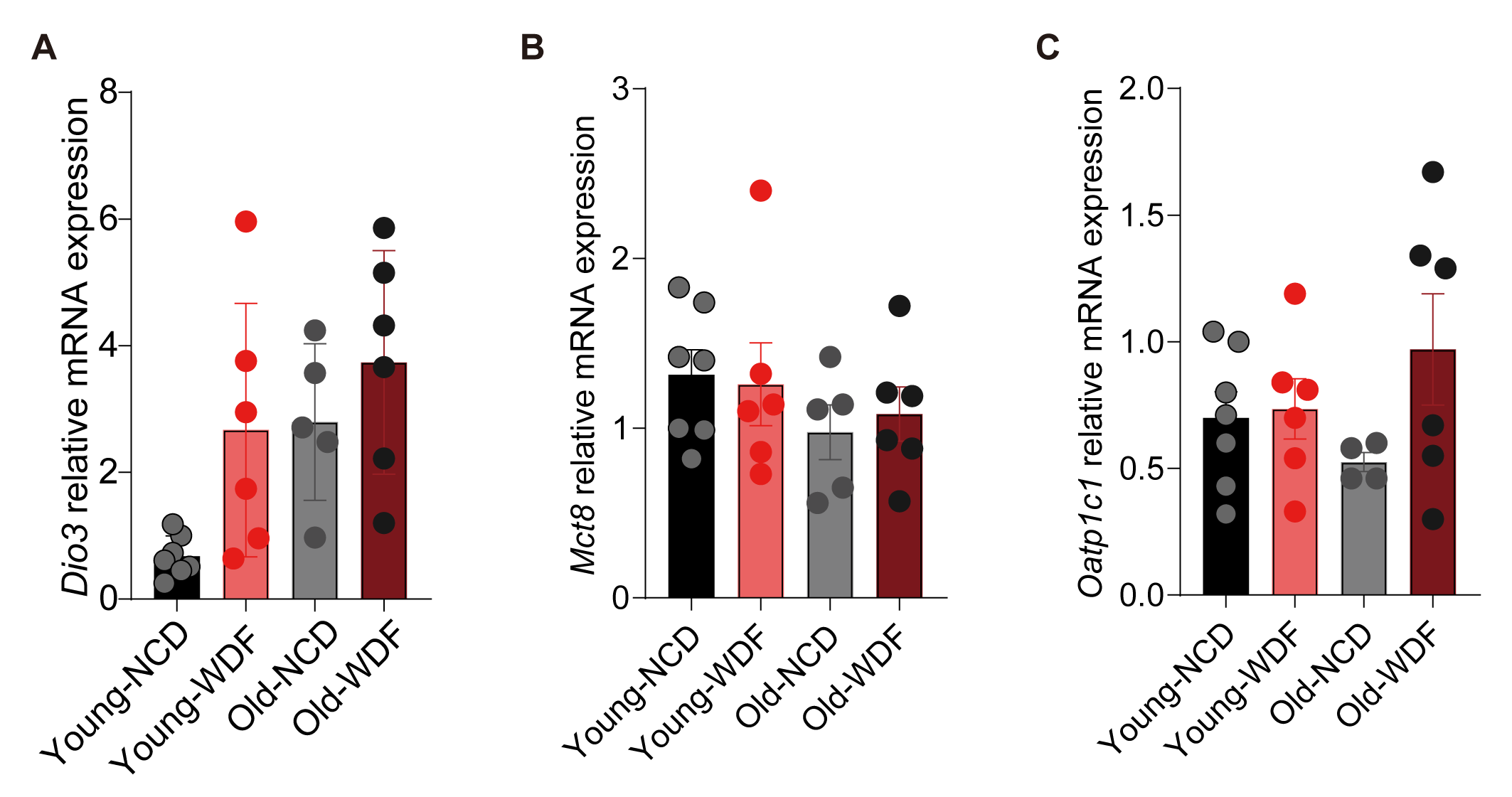

Supplement: Supplementary file 2 — Figure S1: Young (18–24 weeks) and old (108–120 weeks) male mice liver metabolic parameters. (A) Body weight, (B) Fat mass, (C) liver index (liver weight to body weight ratio; LW/BW) and (D) fasting glucose. Statistical analysis was performed by two‐way ANOVA with subsequent Tukey's multiple comparison. Data were presented as mean ± SD. *p < 0.05, **p < 0.01, ***p < 0.001, ****p < 0.0001. Figure S2: Histological assessment of MASLD severity in young and aged mice on NCD or WDF. (A) NAFLD Activity Score (NAS) components and total score in Young‐NCD, Young‐WDF, Old‐NCD, and Old‐WDF mice (n = 5 per group). (B) Representative liver Picrosirius Red staining images showing collagen deposition (10× magnification, scale bar = 100 μm). Data in (A) are presented as mean ± SD with individual data points. Statistical analysis by Kruskal–Wallis test was performed followed by Dunn's multiple comparison test. *p < 0.05, **p < 0.01. Figure S3: Thyroid hormone transporters Mct8 and Oatp1c1 expressions did not change in aging and diet. (A) Dio3 mRNA expressions in young and old mice fed WDF or NCD model. (B, C) Mct8 and Oatp1c1 mRNA expressions in young and old mice fed WDF or NCD model. Thyroid hormone transporters monocarboxylate transporter 8: Mct8, organic anion‐transporting polypeptide 1c1: Oatp1c1. Statistical testing was performed using a two‐way ANOVA with subsequent Tukey's multiple comparison. Data were presented as means ± SD. Figure S4: Young (18–24 weeks) and old (108–120 weeks) male mice liver hedgehog pathway genes. Ihh, Gli3, Angpt1, Sox9, Pcna mRNA expressions in young and old mice fed WDF or NCD model. Statistical analysis was performed by two‐way ANOVA with subsequent Tukey's multiple comparison. Data were presented as mean ± SD. *p < 0.05, **p < 0.01, ***p < 0.001, ****p < 0.0001, ns = no significance. Figure S5: Hepatic Dio1 activity negatively correlated with p21 expression in vivo. Linear regression and correlation analysis between hepatic Dio1 enzyme activity [file ACEL-25-e70600-s005.zip › acel70600-sup-0005-FigureS3-S3@Supplementary_Figure_S3.tif]

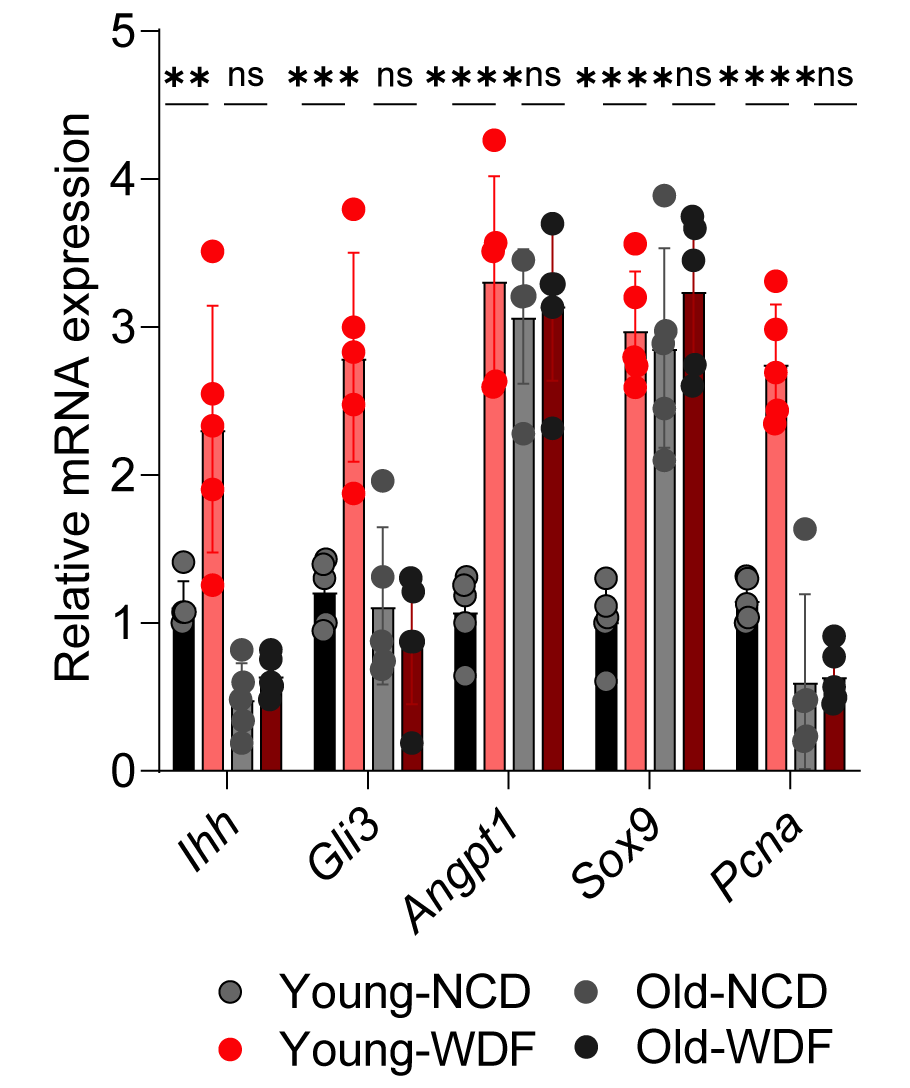

Supplement: Supplementary file 2 — Figure S1: Young (18–24 weeks) and old (108–120 weeks) male mice liver metabolic parameters. (A) Body weight, (B) Fat mass, (C) liver index (liver weight to body weight ratio; LW/BW) and (D) fasting glucose. Statistical analysis was performed by two‐way ANOVA with subsequent Tukey's multiple comparison. Data were presented as mean ± SD. *p < 0.05, **p < 0.01, ***p < 0.001, ****p < 0.0001. Figure S2: Histological assessment of MASLD severity in young and aged mice on NCD or WDF. (A) NAFLD Activity Score (NAS) components and total score in Young‐NCD, Young‐WDF, Old‐NCD, and Old‐WDF mice (n = 5 per group). (B) Representative liver Picrosirius Red staining images showing collagen deposition (10× magnification, scale bar = 100 μm). Data in (A) are presented as mean ± SD with individual data points. Statistical analysis by Kruskal–Wallis test was performed followed by Dunn's multiple comparison test. *p < 0.05, **p < 0.01. Figure S3: Thyroid hormone transporters Mct8 and Oatp1c1 expressions did not change in aging and diet. (A) Dio3 mRNA expressions in young and old mice fed WDF or NCD model. (B, C) Mct8 and Oatp1c1 mRNA expressions in young and old mice fed WDF or NCD model. Thyroid hormone transporters monocarboxylate transporter 8: Mct8, organic anion‐transporting polypeptide 1c1: Oatp1c1. Statistical testing was performed using a two‐way ANOVA with subsequent Tukey's multiple comparison. Data were presented as means ± SD. Figure S4: Young (18–24 weeks) and old (108–120 weeks) male mice liver hedgehog pathway genes. Ihh, Gli3, Angpt1, Sox9, Pcna mRNA expressions in young and old mice fed WDF or NCD model. Statistical analysis was performed by two‐way ANOVA with subsequent Tukey's multiple comparison. Data were presented as mean ± SD. *p < 0.05, **p < 0.01, ***p < 0.001, ****p < 0.0001, ns = no significance. Figure S5: Hepatic Dio1 activity negatively correlated with p21 expression in vivo. Linear regression and correlation analysis between hepatic Dio1 enzyme activity [file ACEL-25-e70600-s005.zip › acel70600-sup-0006-FigureS4-S4@Supplementary_Figure_S4.tif]

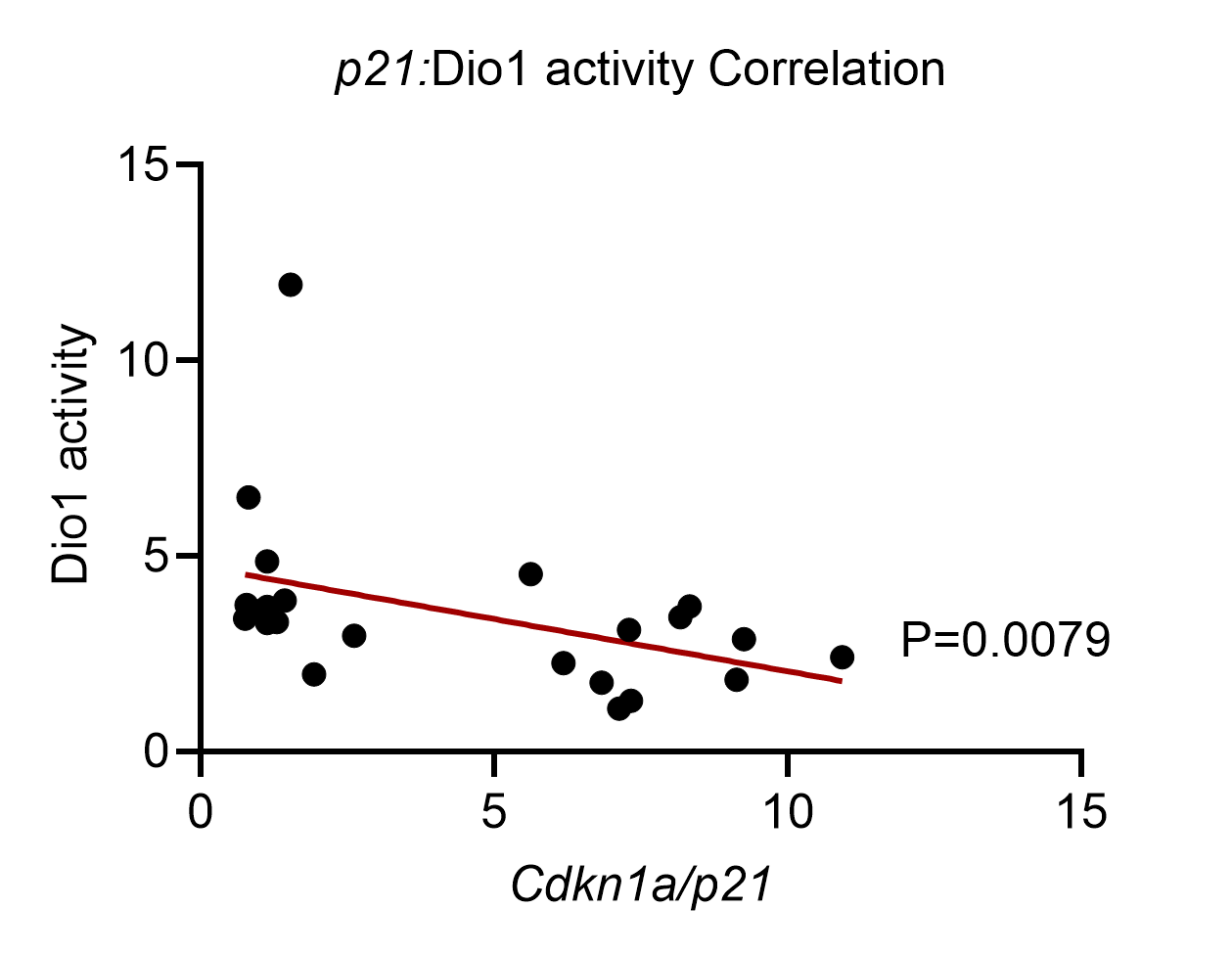

Supplement: Supplementary file 2 — Figure S1: Young (18–24 weeks) and old (108–120 weeks) male mice liver metabolic parameters. (A) Body weight, (B) Fat mass, (C) liver index (liver weight to body weight ratio; LW/BW) and (D) fasting glucose. Statistical analysis was performed by two‐way ANOVA with subsequent Tukey's multiple comparison. Data were presented as mean ± SD. *p < 0.05, **p < 0.01, ***p < 0.001, ****p < 0.0001. Figure S2: Histological assessment of MASLD severity in young and aged mice on NCD or WDF. (A) NAFLD Activity Score (NAS) components and total score in Young‐NCD, Young‐WDF, Old‐NCD, and Old‐WDF mice (n = 5 per group). (B) Representative liver Picrosirius Red staining images showing collagen deposition (10× magnification, scale bar = 100 μm). Data in (A) are presented as mean ± SD with individual data points. Statistical analysis by Kruskal–Wallis test was performed followed by Dunn's multiple comparison test. *p < 0.05, **p < 0.01. Figure S3: Thyroid hormone transporters Mct8 and Oatp1c1 expressions did not change in aging and diet. (A) Dio3 mRNA expressions in young and old mice fed WDF or NCD model. (B, C) Mct8 and Oatp1c1 mRNA expressions in young and old mice fed WDF or NCD model. Thyroid hormone transporters monocarboxylate transporter 8: Mct8, organic anion‐transporting polypeptide 1c1: Oatp1c1. Statistical testing was performed using a two‐way ANOVA with subsequent Tukey's multiple comparison. Data were presented as means ± SD. Figure S4: Young (18–24 weeks) and old (108–120 weeks) male mice liver hedgehog pathway genes. Ihh, Gli3, Angpt1, Sox9, Pcna mRNA expressions in young and old mice fed WDF or NCD model. Statistical analysis was performed by two‐way ANOVA with subsequent Tukey's multiple comparison. Data were presented as mean ± SD. *p < 0.05, **p < 0.01, ***p < 0.001, ****p < 0.0001, ns = no significance. Figure S5: Hepatic Dio1 activity negatively correlated with p21 expression in vivo. Linear regression and correlation analysis between hepatic Dio1 enzyme activity [file ACEL-25-e70600-s005.zip › acel70600-sup-0007-FigureS5-S5@Supplementary_Figure_S5.tif]
