# Supplementary material for: Aging and Western Diet Synergistically Impair Hepatic Thyroid Hormone Signaling to Promote Metabolic Dysfunction‐Associated Steatotic Liver Disease (MASLD) in Mice
Source: Aging Cell. 2026 Jun 23;25(7):e70600. doi: 10.1111/acel.70600 (PMC13288151; doi:10.1111/acel.70600)
Supplement: Supplementary file 3 — Table S1: Two‐way ANOVA results for animal model parameters. [file ACEL-25-e70600-s003.docx]

| **Supplementary Table S1. Two-way ANOVA results for animal model parameters.** | | | | |
| --- | --- | --- | --- | --- |
| Category | Parameter | Age p-value | Diet p-value | Interaction p-value |
| Inflammatory | *Il6* | **<0.0001** | **0.0001** | **0.0264** |
|  | *Il1b* | **0.0146** | **<0.0001** | 0.2730 |
|  | *Tnfa* | **<0.0001** | **0.0447** | 0.6445 |
|  | *Nos2* | **0.0001** | **0.0001** | 0.1395 |
| Fibrosis | *Tgfb1* | **0.0004** | **0.0021** | 0.0588 |
|  | *Col1a1* | **0.0002** | **0.0005** | **0.0048** |
|  | *Acta2* | **0.0002** | **0.0005** | 0.7068 |
|  | *Ctgf* | **<0.0001** | **<0.0001** | **0.0498** |
|  | HPA | **0.0028** | **0.0262** | 0.5287 |
| Senescence | *Cdkn1a/p21* | **<0.0001** | 0.4953 | 0.7246 |
|  | *Cdkn2a/p16* | **<0.0001** | 0.4709 | 0.9121 |
|  | *Tp53* | **<0.0001** | 0.7022 | 0.8329 |
| Intrahepatic TH | T4 | **0.0002** | **<0.0001** | 0.4864 |
|  | T3 | **0.0004** | **<0.0001** | 0.7717 |
| Deiodinase activity | Dio1 activity | **0.0018** | **0.0316** | 0.5063 |
|  | Dio3 activity | **0.0014** | 0.6580 | **0.0033** |
| Deiodinase mRNA | *Dio1* | **0.0251** | **<0.0001** | 0.0770 |
|  | *Dio3* | **0.0150** | **0.0235** | 0.3899 |
| TH transporters | *Mct8* | 0.2699 | 0.7075 | 0.8473 |
|  | *Oatp1c1* | 0.8428 | 0.1243 | 0.1856 |
| Metabolic parameters | Liver index (LW/BW) | **<0.0001** | **<0.0001** | **0.0002** |
|  | Fasting glucose | **0.0005** | **0.0011** | 0.1097 |
|  | Liver TG | 0.1063 | **<0.0001** | 0.3252 |
| Abbreviations: HPA, hydroxyproline; TG, triglyceride; T4, thyroxine; T3, triiodothyronine; Dio1, type 1 deiodinase; Dio3, type 3 deiodinase. | | | | |
