# Supplementary material for: Aging and Western Diet Synergistically Impair Hepatic Thyroid Hormone Signaling to Promote Metabolic Dysfunction‐Associated Steatotic Liver Disease (MASLD) in Mice
Source: Aging Cell. 2026 Jun 23;25(7):e70600. doi: 10.1111/acel.70600 (PMC13288151; doi:10.1111/acel.70600)
Supplement: Supplementary file 4 — Table S2: Kruskal–Wallis test followed by Dunn's multiple comparison test results for NAS. [file ACEL-25-e70600-s001.docx]

| **Supplementary Table S2. Kruskal-Wallis test followed by Dunn’s multiple comparison test results for NAS.** | | | | | |
| --- | --- | --- | --- | --- | --- |
| **NAS** | **Young-NCD vs Young-WDF** | **Young-NCD vs Old-WDF** | **Young-WDF vs Old-WDF** | **Old-NCD vs Old-WDF** | **p-value** |
| Steatosis | **0.0456** | **0.0066** | >0.9999 | **0.0066** | **0.0005** |
| Ballooning | >0.9999 | 0.2394 | 0.2394 | 0.2394 | 0.0965 |
| lobular inflammation | >0.9999 | 0.0792 | >0.9999 | >0.9999 | 0.0815 |
| Total score | 0.0591 | **0.0024** | >0.9999 | 0.1107 | **0.0023** |
| Abbreviations: NAS: NAFLD Activity Score | | | | | |
