# Supplementary material for: Aging and Western Diet Synergistically Impair Hepatic Thyroid Hormone Signaling to Promote Metabolic Dysfunction‐Associated Steatotic Liver Disease (MASLD) in Mice
Source: Aging Cell. 2026 Jun 23;25(7):e70600. doi: 10.1111/acel.70600 (PMC13288151; doi:10.1111/acel.70600)
Supplement: Supplementary file 5 — Table S3: Two‐way ANOVA with subsequent Tukey's multiple comparison results for hedgehog pathway genes. [file ACEL-25-e70600-s002.docx]

| **Supplementary Table S3. Two-way ANOVA with subsequent Tukey’s multiple comparison results for hedgehog pathway genes.** | | | | | |
| --- | --- | --- | --- | --- | --- |
| **Hedgehog pathway** | **Age p-value** | **Diet p-value** | **Interaction p-value** | **Young-NCD vs Young-WDF** | **Old-NCD vs Old-WDF** |
| *Ihh* | **<0.0001** | **0.0037** | **0.0197** | **0.0032** | 0.9403 |
| *Ptch1* | **<0.0001** | 0.5547 | **0.0061** | 0.0730 | 0.3057 |
| *Bcl2* | 0.0524 | **0.0471** | 0.7199 | 0.3186 | 0.5981 |
| *Gli3* | **0.0004** | **0.0086** | **0.0011** | **0.0008** | 0.8940 |
| *Hhip* | 0.6132 | **<0.0001** | 0.5616 | **0.0011** | **0.0059** |
| *Angpt1* | **0.0010** | **0.0001** | **0.0002** | **<0.0001** | 0.9954 |
| *Smo* | 0.6746 | **<0.0001** | 0.2494 | **0.0002** | **<0.0001** |
| *Sox9* | **0.0002** | **<0.0001** | **0.0024** | **<0.0001** | 0.6137 |
| *Spp1* | 0.1189 | **0.0152** | 0.1436 | **0.0376** | 0.8376 |
| *Jag1* | **0.0012** | **0.0225** | 0.1684 | 0.0556 | 0.8690 |
